# Complement Component C1q as an Emerging Biomarker for the Diagnosis of Tuberculous Pleural Effusion

**DOI:** 10.3389/fmicb.2021.765471

**Published:** 2021-11-01

**Authors:** Xin Qiao, Ming-Ming Shao, Feng-Shuang Yi, Huan-Zhong Shi

**Affiliations:** Department of Respiratory and Critical Care Medicine, Clinical Center for Pleural Diseases, Beijing Institute of Respiratory Medicine and Beijing Chao-Yang Hospital, Capital Medical University, Beijing, China

**Keywords:** c1q, tuberculous pleural effusion, diagnosis, age, biomarker

## Abstract

**Background and Objective:** The accurate differential diagnosis of tuberculous pleural effusion (TPE) from other exudative pleural effusions is often challenging. We aimed to validate the accuracy of complement component C1q in pleural fluid (PF) in diagnosing TPE.

**Methods:** The level of C1q protein in the PF from 49 patients with TPE and 61 patients with non-tuberculous pleural effusion (non-TPE) was quantified by enzyme-linked immunosorbent assay, and the diagnostic performance was assessed by receiver operating characteristic (ROC) curves based on the age and gender of the patients.

**Results:** The statistics showed that C1q could accurately diagnose TPE. Regardless of age and gender, with a cutoff of 6,883.9 ng/mL, the area under the curve (AUC), sensitivity, specificity, positive predictive value (PPV), and negative predictive value (NPV) of C1q for discriminating TPE were 0.898 (95% confidence interval: 0.825–0.947), 91.8 (80.4–97.7), 80.3 (68.2–89.4), 78.9 (69.2–86.2), and 92.5 (82.6–96.9), respectively. In subgroup analysis, the greatest diagnostic accuracy was achieved in the younger group (≤ 50 years of age) with an AUC of 0.981 (95% confidence interval: 0.899–0.999) at the cutoff of 6,098.0 ng/mL. The sensitivity, specificity, PLR, NLR, PPV, and NPV of C1q were 95.0 (83.1–99.4), 92.3 (64.0–99.8), 97.4 (85.2–99.6), and 85.7 (60.6–95.9), respectively.

**Conclusion:** Complement component C1q protein was validated by this study to be a promising biomarker for diagnosing TPE with high diagnostic accuracy, especially among younger patients.

## SUMMARY AT A GLANCE

Our study is the first to investigate the diagnostic efficacy of C1q in pleural fluid in differentiating TPE from non-TPE according to patients’ age and gender.

## Introduction

Tuberculosis (TB) resulted from Mycobacterium tuberculosis (M*tb*) infection is known as one of the major causes of death worldwide. Tuberculous pleural effusion (TPE) is the most common extrapulmonary form of TB (Baumann et al., 2007; Peto et al., 2009; Pang et al., 2019; Kang et al., 2020). Although TPE is clinically common, its differentiation from other types of exudative pleural effusion, such as malignant pleural effusion (MPE), parapneumonic pleural effusion (PPE), and other types of pleural effusion due to autoimmune diseases etc., is often challenging. Smear microscopy with Ziehl–Neelsen stains, M*tb* culture, and pleural biopsy are the gold standards for the diagnosis of TPE; however, the positive rates of Ziehl–Neelsen staining and M*tb* culture are both very low. Pleural biopsy is an invasive procedure with the concerns that the complications associated with surgery that cannot be ignored (Wang et al., 2015). Compared with the above methods, biomarkers in pleural fluid (PF), such as adenosine deaminase (ADA), are an affordable, simplified, non-invasive, and rapid diagnostic method for TPE (Porcel, 2016; Zhang et al., 2020).

Recently, complement has been highlighted as a candidate biomarker for active TB. There are a few studies on the role of C1q in TB immunity that have suggested C1q to be useful in the differential diagnosis of human activity infection and latent infection of M*tb* (Cai et al., 2014; Lubbers et al., 2018), although most have focused on the C1q level in serum. In addition, some studies on TPE have enrolled patients with transudative pleural effusion, which can be separated effectively from exudative effusions using Light’s criteria (Cai et al., 2014; Luo et al., 2019). Meanwhile, age and gender have important effects on the immune system (Markle and Fish, 2014; Giefing-Kröll et al., 2015), which may affect the diagnostic accuracy of diagnostic markers (Jiang et al., 2020). Therefore, we conducted this study to identify the exact role of complement component C1q in the diagnosis of TPE according to age and gender.

## Materials and Methods

### Study Populations and Sample Collection

Consecutive pleural effusion patients were enrolled in the Department of Respiratory and Critical Care Medicine, Beijing Chao-yang Hospital, Capital Medical University, between April 2019 and October 2020. Patients who underwent any invasive pleural surgery or experienced chest trauma during the 3 months prior to their hospitalization; who had received any anti-TB chemotherapy, antitumor treatment, glucocorticoids, or other non-steroidal anti-inflammatory therapy were excluded. Due to the activation of complement system is related to the pathogenesis of diabetes (Shim et al., 2020). This may cause complicated changing of the level of C1q in pleural fluid, lead to unpredictable results, we excluded the patients complicated with diabetes. A total of 110 patients with a definite diagnosis of exudative pleural effusion were included in our study. Non-TPE cases included patients with MPE, PPE, or various pleural effusion. TPE was diagnosed if Ziehl–Neelsen staining or M*tb* culture of PF or pleural biopsy specimens were positive, or if a granuloma was present in the pleural biopsy specimens. MPE was diagnosed when malignant cells were observed in PF and/or pleural biopsy specimens. PPE was diagnosed as any effusions related to bacterial pneumonia, lung abscess, and bronchiectasis with infection. The remaining effusions consisted of exudates caused by coronary artery bypass surgery or autoimmune diseases. Patients’ baseline data are illustrated in Table 1.

**TABLE 1:** Baseline characteristics according to study population.

**Variable**	**Total population**	**TPE (*n* = 49)**	**Non-TPE (*n* = 61)**	***P*-value**
Gender, male/female, n	75/35	32/17	43/18	0.562
Age, years	51 (31, 71)	31 (24, 43)	65 (51, 77)	< 0.001
Total cell count × 10^9^/L	4.7 (2.1, 16.0)	4.1 (2.3, 6.8)	9.5 (2.1, 72.6)	0.007
Protein, g/L	45.6 ± 9.1	50.3 ± 6.3	41.8 ± 9.3	< 0.001
LDH, U/L	340.5 (223.3, 594.0)	444.0 (320.0, 628.5)	279.0 (174.5, 472.5)	0.003
Cl^–^, mmol/L	106.0 (103.1, 108.7)	104.9 (101.6, 107.2)	106.8 (104.1, 109.5)	0.007
Glucose, mmol/L	5.2 ± 2.3	4.6 ± 1.6	5.7 ± 2.7	0.007

*Data are presented as mean ± SD or median (25th–75th centile). Differences of continuous data between groups were compared using Student’s t-test or Mann-Whitney U-test, and χ^2^-test was used for comparisons of categorical data. TPE, tuberculous pleural effusion, non-TPE, non-tuberculous pleural effusion.*

PF was collected by diagnostic thoracentesis from each patient. At the same time, peripheral blood was also collected, and the PF and paired blood samples were quickly transferred to the laboratory at 4°C and centrifuged at 400 g for 10 min at 4°C. The supernatant was stored at −80°C for later C1q and ADA measurement.

This study was approved by the ethics committees of Beijing Chao-yang Hospital, Capital Medical University, and all participants had written informed consent.

### Concentration Determination of C1q Protein

The concentration of C1q protein in PF and plasma were tested by enzyme-linked immunosorbent assay (ELISA) kits with reference to the manufacturer’s specifications (Thermo Fisher Scientific, Waltham, MA, United States). The level of ADA was determined using colorimetric method kits (InTec Products, Inc., Xiamen, China) in accordance with the manufacturer’s specifications. All samples were assayed in duplicate.

### Statistical Analysis

Continuous statistics were expressed as mean ± standard deviation or medians (25th–75th centiles). Categorical data were described by frequencies. Differences in continuous statistics between groups were compared using Student’s *t-*test or Mann-Whitney *U*-test, while the χ^2^-test was used for comparing categorical data. Receiver operating characteristic (ROC) analysis was applied to identify the power of C1q to distinguish TPE and non-TPE cases, and results were presented as area under the Curve (AUC) (Hanley and McNeil, 1982; Zweig and Campbell, 1993). Statistical analyses were performed using SPSS and MedCalc software, and statistical significance was present when *P* < 0.05.

## Results

### Clinical and Demographic Characteristics of Patients With Pleural Effusion

Some biochemical, cytological, and demographic data of the TPE and non-TPE patient groups are illustrated in Table 1. According to our preliminary statistical results, we selected the age of 50 years as the cutoff for the age subgroups: younger group (≤ 50 years old) and older group (> 50 years old), respectively. In this study, TPE patients were younger than non-TPE patients (*P* < 0.001). The total cell count, Cl- level, and glucose level in TPE patients were lower than those in non-TPE patients (all *P* = 0.007). Compared with those in non-TPE patients, protein and lactate dehydrogenase (LDH) levels in TPE patients were significantly higher (*P* < 0.001 and *P* = 0.003, respectively).

### Concentrations of C1q and as Adenosine Deaminase in Pleural Fluid

**FIGURE 1:**
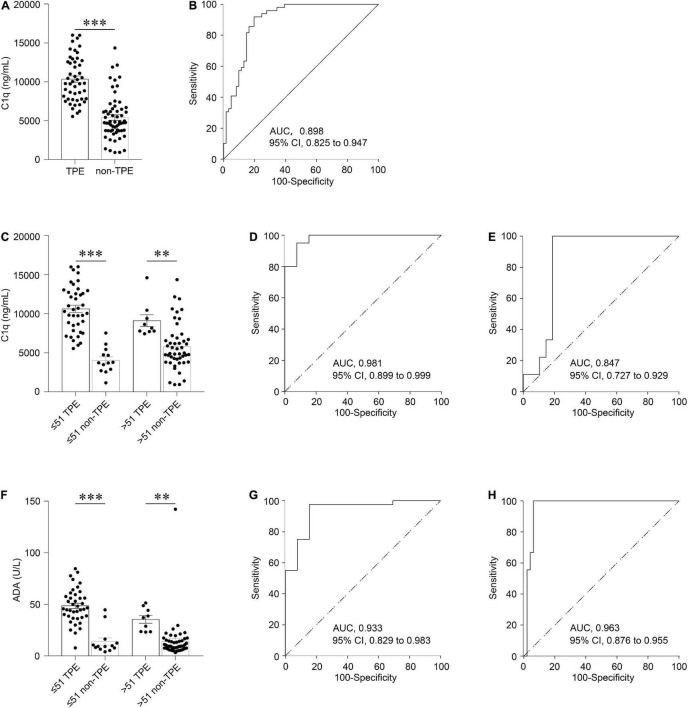
Diagnostic accuracy of C1q and ADA in PF for TPE according to different ages. Comparison of C1q level in TPE and those in non-TPE cases **(A)**. The ROC curves show the diagnostic value of C1q in all patients **(B)**. Comparison of C1q in TPE and those in non-TPE cases according to age **(C)**. The ROC curves show the diagnostic value of C1q in younger patients **(D)** and older patients **(E)**, respectively. Comparison of ADA in TPE and those in non-TPE cases according to age **(F)**. The ROC curves show the diagnostic value of ADA in younger patients **(G)** and older patients **(H)**, respectively. ^∗∗^*P* < 0.01, ^∗∗∗^*P* < 0.001.

Regardless of age and gender, the levels of C1q and ADA were higher in the TPE group than in the non-TPE group (*P* < 0.001) (Table 2, Figure 1A, and Supplementary Figure 1D). Overall, the concentration of C1q was lower in PF than in plasma (*P* < 0.001). In addition, plasma concentrations of C1q in TPE patients did not differ from those in non-TPE patients (*P* = 0.965) (Supplementary Table 1). Similar statistical differences were found in the different age subgroups (Table 2 and Figures 1C,F) and gender subgroups (Supplementary Table 2 and Supplementary Figures 1A,F).

**TABLE 2:** Concentrations of C1q and ADA in PF according to age.

**Variable**	**TPE**	**Non-TPE**	***P*-value**
C1q, ng/mL	10331.4 ± 2882.1	5418.5 ± 2819.4.	<0.001
Age ≤ 50 y	10608.2 ± 2955.4	4034.5 ± 1669.2	<0.001
Age > 50 y	9101.4 ± 2274.4	5793.4 ± 2960.2	0.002
ADA, U/L	44.7 (36.4, 56.0)	9.4 (7.4, 15.0)	<0.001
Age ≤ 50 y	46.1 (37.6, 59.0)	9.3 (7.1, 14.3)	<0.001
Age > 50 y	37.2 (23.5, 46.4)	9.4 (7.3, 15.1)	<0.001

*Data are presented as mean ± SD or median (25th–75th centile). Differences between groups were compared using Student’s t-test for C1q or Mann-Whitney U-test for ADA. TPE, tuberculous pleural effusion, non-TPE, non-tuberculous pleural effusion.*

### Diagnostic Values of Pleural Fluid C1q and as Adenosine Deaminase

In the general patients, with a cutoff of 6,883.9 ng/mL, the AUC, sensitivity, specificity, positive likelihood ratio (PLR), negative likelihood ratio (NLR), positive predictive value (PPV), and negative predictive value (NPV) of PF C1q to discriminate TPE and non-TPE cases were 0.898 (95% confidence interval: 0.825–0.947; P < 0.001), 91.8%, 80.3%, 4.7, 0.10, 78.9, and 92.5, respectively (Figure 1B and Table 3). Meanwhile, the AUC of ADA was 0.953 (Supplementary Figure 1E), and there was no significant difference in the AUCs between C1q and ADA (0.055 (−0.015 to 0.125), *z* = 1.539, *P* = 0.124).

**TABLE 3:** Diagnostic performance of C1q and ADA in PF in differentiating between patients with TPE and those with non-TPE according to age.

**Variable**	**Cut-off value (ng/mL)**	**AUC (95% CI)**	**Sensitivity (%)**	**Specificity (%)**	**PLR**	**NLR**	**PPV**	**NPV**
C1q	6883.9	0.898	91.8	80.3	4.7	0.10	78.9	92.5
		(0.825–0.947)	(80.4–97.7)	(68.2–89.4)	(2.8–7.8)	(0.04–0.30)	(69.2–86.2)	(82.6–96.9)
Age ≤ 50 y	6098.0	0.981	95.0	92.3	12.4	0.05	97.4	85.7
		(0.899–0.999)	(83.1–99.4)	(64.0–99.8)	(1.9–81.3)	(0.01–0.20)	(85.2–99.6)	(60.6 –95.9)
Age > 50 y	7395.7	0.847	100.0	81.3	5.3	0.00	50.0	100.0
		(0.727–0.929)	(66.4–100.0)	(67.4–91.1)	(3.0–9.6)	−	(35.7–64.3)	
ADA	22.01	0.953	98.0	90.2	9.96	0.02	88.9	98.2
		(0.895–0.984)	(89.1–99.9)	(79.8–96.3)	(4.7–21.3)	(0.00–0.20)	(78.9– 94.5)	(88.8–99.7)
Age ≤ 50 y	16.30	0.933	97.5	84.6	6.34	0.03	95.1	84.6
		(0.829–0.983)	(86.8–99.9)	(54.6–98.1)	(1.8–22.7)	(0.00–0.20)	(84.5–98.6)	(58.3–95.6)
Age > 50 y	22.49	0.963	100.0	93.8	16.0	0.00	75.0	100.0
		(0.876–0.995)	(66.4–100.0)	(82.8–98.7)	(5.3–47.9)	−	(50.1–90.0)	−

*AUC, area under the curve; PLR, positive likelihood ratio; NLR, negative likelihood ratio; PPV, positive predictive value; NPV, negative predictive value.*

The measures of diagnostic accuracy of PF C1q in different subgroups were also determined. With a cutoff of 6,098.0 ng/mL, the AUC of PF C1q in the younger group to differentiate TPE and non-TPE cases was 0.981 (95% confidence interval: 0.899–0.999; *P* < 0.001), while the sensitivity, specificity, PLR, NLR, PPV, and NPV of C1q were 95.0%, 92.3%, 12.4, 0.05, 97.4, and 85.7, respectively (Figure 1D and Table 3). The older group, male group, and female group had AUCs of 0.847 (0.727–0.929) (Figure 1E and Table 3), 0.922 (0.836–0.971) (Supplementary Figure 1B and Supplementary Table 3), and 0.827 (0.661–0.933) (Supplementary Figure 1C and Supplementary Table 3), respectively. Further, the parameters of diagnostic accuracy of ADA in the different subgroups are shown in Table 3, Figures 1G,H, Supplementary Table 3, and Supplementary Figures 1G,H.

The AUC of C1q in younger group was significantly higher than that in older group (0.134 (0.028–0.240); *z* = 2.468; *P* = 0.014); however, there was no significant difference between younger patients and older patients in terms of ADA [0.030 (−0.064 to 0.124), *z* = 0.631, *P* = 0.528). There was no difference found in the gender subgroup analysis of C1q and ADA [0.09 (−0.057 to 0.246), *z* = 1.224, *P* = 0.221 and 0.113 (−0.024 to 0.250), *z* = 1.613, *P* = 0.107)].

## Discussion

According to the global tuberculosis report 2020 (WHO, 2020), there were about 10.0 million people had been diagnosed with TB in 2019. According to the data in the report, as one of the countries with high burden of TB, there were approximately 833,000 people fell ill with TB in China in 2019, and the case fatality rate was about 4.0%. Extrapulmonary TB accounted for 16% of the incident cases. The pleura has been reported as the major site of disease in patients with extrapulmonary TB, and tuberculous pleurisy occurs in approximately 50% of patients with extrapulmonary TB (Kang et al., 2020). Early animal studies have shown that tuberculous pleurisy is thought to be a delayed hypersensitivity reaction induced by a small amount of M*tb* entering the thoracic cavity rather than a local inflammatory reaction caused by direct infection (Allen and Apicella, 1968; Leibowitz et al., 1973; Chakrabarti and Davies, 2006; Zhai et al., 2016). Therefore, the bacillary load in pleural effusion is low, and the diagnosis of TPE is often challenging to make and sometimes requires invasive surgery to obtain pleural tissue for histological and microbiological examinations (Amer et al., 2016; Antonangelo et al., 2019). Most cases of TPE can be diagnosed through medical thoracoscopy (Wang et al., 2015; Carlucci et al., 2019). However, not everyone has the indication to undergo a medical thoracoscopy nor is everyone willing to undergo this examination. Therefore, some soluble biomarkers in PF have been extensively evaluated (Porcel, 2016; Wang et al., 2018; Zhang et al., 2020).

The complement system consists of more than 50 kinds of proteins that either circulate in the fluid phase or bind to the cell membrane, which has varied effector functions and plays an important role in both innate and adaptive immune responses (West et al., 2018; Conigliaro et al., 2019; Shim et al., 2020). When M*tb* is inhaled into the lungs of human hosts for the first time, it will activate the complement classical pathway in the alveoli (Ferguson et al., 2004). Complement C1q is the first recognition subunit of the complement classical pathway whose gene polymorphism is closely related to TB susceptibility (Bruiners and Schurz, 2020). Research has suggested C1q can be used as a soluble mediator to evaluate TB progression in primates (Dijkman et al., 2020). Both C1q gene expression on mononuclear cells in peripheral blood and the serum C1q protein levels are related to active disease in human tuberculosis (Cai et al., 2014; Lubbers et al., 2018), and there is a progressive decrease in plasma C1q mRNA expression and plasma C1qC protein during anti-tuberculosis chemotherapy (Cai et al., 2014). In our study, no difference was found in plasma C1q levels between TPE and non-TPE patients, which may have been caused by variations in disease stages of enrolled participants in previous studies and our study. Similar to previous findings (Cai et al., 2014; Luo et al., 2019), through this study, we confirmed that C1q protein levels in TPE patients were remarkably higher than those in non-TPE patients, the cause of this issue maybe similar to the cases of pulmonary tuberculosis we mentioned above. The diagnostic efficacy of C1q was comparable to that of ADA, the preferred soluble biomarker for TPE (Zhang et al., 2020). C1q is a great diagnostic biomarker for discriminating TPE and non-TPE cases, and we further conducted subgroup analysis according to age and gender.

The contributions of age and gender to an immune response are significant (Pawelec, 2006; Markle and Fish, 2014). There is a significant postpubertal male bias in the incidence of TB (Guerra-Silveira and Abad-Franch, 2013). Meanwhile, although the serum complement C1q concentrations do not differ between healthy females and males, the activity of the complement classical pathway and the circulating complement C1q level are significantly higher in the elderly population (Gaya da Costa et al., 2018; Hasegawa et al., 2019). However, few studies have examined the influencing factors of pleural soluble mediators for TPE diagnosis, especially in correlation with age and gender (Abrao et al., 2014; Jiang et al., 2020). Our results revealed that the concentration of PF C1q did not differ between younger and older patients or between male and female patients. Compared with in the older group, the AUC of PF C1q in the younger group was significantly higher, but there was no statistical difference between male group and female group. Meanwhile, no significant differences in the level of ADA were found among the age or gender subgroups. At present, to our knowledge, this study is the first to investigate the diagnostic efficacy of PF C1q level in differentiating TPE from non-TPE according to patients’ age and gender.

For PF C1q in younger patients, our data also discerned a PLR value of 12.4, indicating that the probability of positive C1q in TPE patients was 12.4-fold higher than that in non-TPE patients, which is sufficiently high enough for diagnosis. Moreover, an NLR value of 0.05 suggested that, if the C1q result is negative, the probability of the patient be confirmed to have TPE was 5%, which was an acceptable value for ruling out TPE. The high PPV (97.4) and high NPV (85.7) of C1q found in this study further indicate that both the false-negative and false-positive rates were low.

There are some restrictions in this research. First, the numbers of participants enrolled in this study were relatively small, especially those who were eligible for inclusion in the younger non-TPE group, the older TPE group, and the female group. According to epidemiology, the incidence of non-TPE in younger patients and the incidence of female pleural effusion are relatively low, and a small sample size may be influenced by selection bias. Second, most of the MPE cases analyzed in our study were derived from lung adenocarcinoma, and non-TPE cases caused by autoimmune diseases were rare. We aim to pursue the analysis of pleural effusion caused by other types of malignant and benign diseases and confirm the mechanism related to PF C1q in our further studies.

In conclusion, our present data indicated that the level of PF C1q was increased in TPE patients compared with non-TPE patients. Both of the sensitivity and specificity of PF C1q were high, suggesting that it can be used as an indicator for differentiating TPE and non-TPE, especially in younger patients.

## Data Availability Statement

The original contributions presented in the study are included in the article/Supplementary Material, further inquiries can be directed to the corresponding author/s.

## Ethics Statement

The studies involving human participants were reviewed and approved by the Ethics Committees of Beijing Chao-yang Hospital, Capital Medical University. The patients/participants provided their written informed consent to participate in this study.

## Author Contributions

XQ, M-MS, and F-SY designed and conducted most of the experiments, did patient recruitment and assessment, collected information, and analyzed the relevant data. H-ZS and F-SY conceived the idea, guided the study, and critically revised the manuscript to ensure the integrity of this research. All authors read, critically revised, and approved the final manuscript.

## Conflict of Interest

The authors declare that the research was conducted in the absence of any commercial or financial relationships that could be construed as a potential conflict of interest.

## Publisher’s Note

All claims expressed in this article are solely those of the authors and do not necessarily represent those of their affiliated organizations, or those of the publisher, the editors and the reviewers. Any product that may be evaluated in this article, or claim that may be made by its manufacturer, is not guaranteed or endorsed by the publisher.
