# Supplementary material for: Complement Component C1q as an Emerging Biomarker for the Diagnosis of Tuberculous Pleural Effusion
Source: Front Microbiol. 2021 Nov 1;12:765471. doi: 10.3389/fmicb.2021.765471 (PMC8591783; doi:10.3389/fmicb.2021.765471)
Supplement: Supplementary file 1 [file Data_Sheet_1.docx]

**Complement component C1q as an emerging biomarker for the diagnosis of tuberculous pleural effusion**

**Xin Qiao, Ming-Ming Shao, Feng-Shuang Yi, Huan-Zhong Shi**

Department of Respiratory and Critical Care Medicine, Beijing Institute of Respiratory Medicine and Beijing Chao-Yang Hospital, Capital Medical University

**Figure S1**
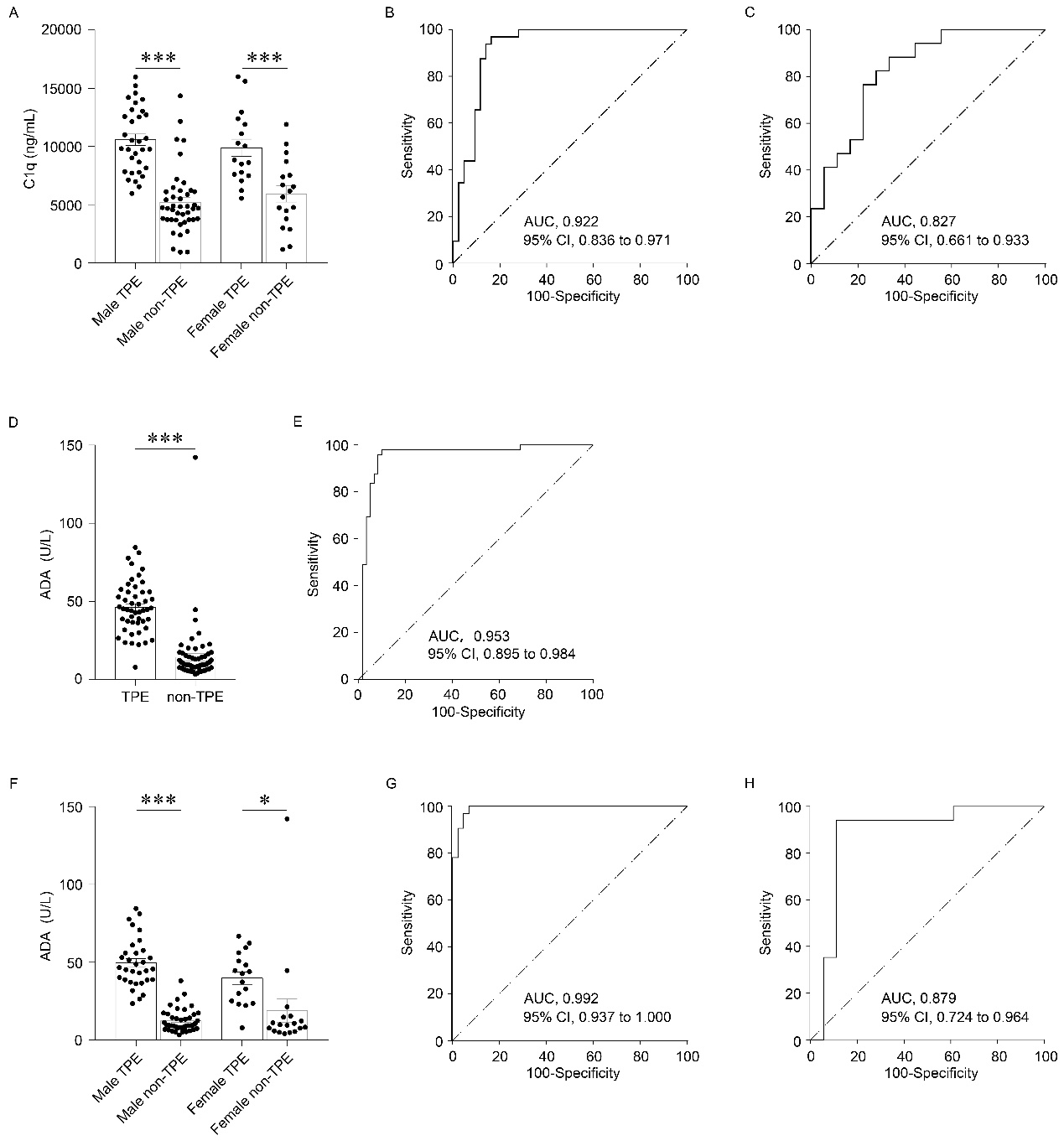


Diagnostic accuracy of C1q and ADA in PF for TPE according to different genders. Comparison of C1q level in TPE and those in non-TPE cases according to gender (A). The ROC curves show the diagnostic value of C1q in male patients (B) and in female patients (C), respectively. Comparison of ADA in TPE and those in non-TPE (D). The ROC curves show the diagnostic value of ADA in all patients (E). Comparison of ADA in TPE and those in non-TPE cases according to gender (F). The ROC curves show the diagnostic value of ADA in male patients (G) and in female patients (H), respectively. *P < 0.05, **P < 0.01, ***P < 0.001

**Table S1.** Concentrations of C1q in plasma according to age and gender

| Variable | TPE | Non-TPE | P value |
| --- | --- | --- | --- |
| C1q, ng/mL | 11296.9 ± 2089.0 | 11251.7 ± 3982.1 | 0.965 |
| Age≤50 y | 11426.5 ± 2364.1 | 11837.1 ± 3740.5 | 0.774 |
| Age>50 y | 10908.2 ± 956.2 | 11042.6 ± 4180.0 | 0.945 |
| male | 11033.4 ± 1840.6 | 11994.8 ± 4370.0 | 0.475 |
| female | 11786.3 ± 2571.7 | 9641.6 ± 2589.5 | 0.163 |

Data are presented as mean ± SD. Comparisons of data between TPE and non-TPE were performed using Student's *t*-test.

**Table S2** Concentrations of C1q and ADA in PF according to gender

| Variable | TPE | Non-TPE | P value |
| --- | --- | --- | --- |
| C1q, ng/mL |  |  |  |
| male | 10571.3±2793.7 | 5208.3±2759.0 | <0.001 |
| female | 9880.0±3076.6 | 5920.7±2978.4 | <0.001 |
| ADA, U/L |  |  |  |
| male | 46.1 (38.6, 57.2) | 9.4 (7.5, 16.3) | <0.001 |
| female | 42.7 (24.3, 53.4) | 9.6 (5.7, 14.9) | <0.001 |

Data are presented as mean ± SD or median (25th - 75th centile). Differences between groups were compared using Student's *t*-test for C1q or Mann-Whitney *U* test for ADA. TPE = tuberculous pleural eﬀusion, non-TPE = non-tuberculous pleural eﬀusion.

**Table S3** Diagnostic performance of C1q and ADA in PF in differentiating between patients with TPE and those with non-TPE according to gender

| Variable | Cut-off value (ng/mL) | AUC  (95% CI) | Sensitivity  (%) | Specificity  (%) | PLR | NLR | PPV | NPV |
| --- | --- | --- | --- | --- | --- | --- | --- | --- |
| C1q |  |  |  |  |  |  |  |  |
| male | 6480.9 | 0.922  (0.836 to 0.971) | 96.9  (83.8 to 99.9) | 83.7  (69.3 to 93.2) | 6.0(3.0 to 11.8) | 0.04  (0.01 to 0.30) | 81.6  (69.2 to 89.7) | 97.3  (83.9 to 99.6) |
| female | 6696.7 | 0.827  (0.661 to 0.933) | 88.2  (63.6 to 98.5) | 66.7  (41.0 to 86.7) | 2.7(1.3 to 5.2) | 0.18  (0.05 to 0.70) | 71.4  (56.0 to 83.1) | 85.7  (61.1 to 95.8) |
| ADA |  |  |  |  |  |  |  |  |
| male | 22.49 | 0.992  (0.937 to 1.000) | 100.0  (89.1 to 100.0) | 93.0  (80.9 to 98.5) | 14.3(4.8 to 42.7) | 0.00  - | 91.4  (78.2 to 96.9) | 100.0  - |
| female | 21.31 | 0.879  (0.724 to 0.964) | 94.1  (71.3 to 99.9) | 88.9  (65.3 to 98.6) | 8.5(2.3 to 31.5) | 0.01  (0.01 to 0.40) | 88.9  (68.3 to 96.7) | 94.1  (70.4 to 99.1) |

AUC = area under the curve, PLR = positive likelihood ratio, NLR = negative likelihood ratio, PPV = positive predictive value, NPV = negative predictive value
